# Effects of distinctive encoding on correct and false memory:A meta-analytic review of costs and benefits and their origins in the DRM paradigm

**DOI:** 10.3758/s13423-014-0648-8

**Published:** 2014-05-23

**Authors:** Mark J. Huff, Glen E. Bodner, Jonathan M. Fawcett

**Affiliations:** 1 Department of Psychology, Washington University in St. Louis, St. Louis, MO 63130 USA; 2 University of Calgary, Calgary, AB Canada; 3 MRC Cognition and Brain Sciences Unit, Cambridge, UK

**Keywords:** Recognition, Distinctiveness, DRM paradigm, Encoding, Retrieval, Meta-analysis

## Abstract

We review and meta-analyze how distinctive encoding alters encoding and retrieval processes and, thus, affects correct and false recognition in the Deese–Roediger–McDermott (DRM) paradigm. Reductions in false recognition following distinctive encoding (e.g., generation), relative to a nondistinctive read-only control condition, reflected both impoverished relational encoding and use of a retrieval-based distinctiveness heuristic. Additional analyses evaluated the costs and benefits of distinctive encoding in within-subjects designs relative to between-group designs. Correct recognition was design independent, but in a within design, distinctive encoding was less effective at reducing false recognition for distinctively encoded lists but more effective for nondistinctively encoded lists. Thus, distinctive encoding is not entirely “cost free” in a within design. In addition to delineating the conditions that modulate the effects of distinctive encoding on recognition accuracy, we discuss the utility of using signal detection indices of memory information and memory monitoring at test to separate encoding and retrieval processes.

## Introduction

The finding that distinctively encoded information enjoys a memory advantage has a rich history in memory research (for reviews, see Hunt and Worthen 2006; Schmidt 1991). Nearly any memory benefit for unique stimuli or for stimuli studied in unique ways, relative to control stimuli or encoding tasks, has been attributed to distinctive processing, including effects of isolation (Kelley and Nairne 2001; von Restorff 1933), levels of processing (Craik and Lockhart 1972), generation (Slamecka and Graf 1978), production (Hopkins and Edwards 1972; MacLeod et al. 2010), item-specific processing (Hunt and Einstein 1981), and bizarreness (McDaniel and Einstein 1991). The widely applied construct of distinctiveness has been defined as the “processing of difference in the context of similarity” (Hunt 2006, p. 12). This definition of the phenomenon wisely steers clear of specifying both the processes underlying distinctiveness effects and whether they emerge at study and/or at test. Our review uses a meta-analytic approach to examine the contributions of encoding and retrieval processes to distinctiveness effects in both correct memory and false memory errors. To this end, we focused on a commonly used and powerful false memory paradigm—the Deese–Roediger–McDermott (DRM) paradigm (Deese 1959; Roediger and McDermott 1995; for reviews, see Gallo 2006, 2010). Our findings confirm that distinctive encoding can improve correct memory while reducing false memory but also reveal that these benefits can come with costs. Moreover, we show that the pattern of costs/benefits depends on whether distinctive (vs. nondistinctive) encoding is varied within a group or between groups.

As reviewed by Hunt (2006), it seems intuitive that distinctive (vs. nondistinctive) encoding would require more attention and/or processing, suggestive of an encoding-based mechanism (Jenkins and Postman 1948; McDaniel and Geraci 2006). Indeed, an encoding locus for distinctiveness effects is supported by studies of divided attention at study, a manipulation that curtails participants’ ability to engage in distinctive processing. Consistent with an encoding locus, dividing attention at study reduces the advantage for orthographically distinct (vs. regular) items in recall (Geraci and Rajaram 2002), and for low- (vs. high-) frequency words in recognition (Joordens and Hockley 2000).

Other evidence, however, suggests that distinctive encoding can benefit memory at retrieval. Von Restorff (1933) had participants study a list in which one item was isolated on a particular dimension, relative to others (e.g., a syllable within a list of digits). At test, recall was greater for the isolated item than for the nonisolated items. Importantly, this isolation effect occurred even when the isolate was presented at the start of the list before a context could have been established to contrast with the distinctive isolate (see also Dunlosky et al. 2000). Therefore, distinctiveness at encoding could not have produced this isolation benefit. Instead, the first-position isolate could be perceived as distinct only after the isolate was encoded, at which point the list context could be used during test to provide diagnostic evidence that the isolate was studied (Gallo 2004; Waddill and McDaniel 1998). This argument assumes that encoding of the isolate ends with the presentation of subsequent list items, but participants may return to rehearsing the isolate once the following items have established a context. This could result in the retrieval and preferential rehearsal of the isolate—to the detriment of other list items—suggesting that encoding might still contribute to distinctiveness effects. If so, whether the isolation effect reflects an encoding or a retrieval process becomes difficult to separate and resolve.

In addition to improving correct memory, distinctive encoding has also been shown to substantially reduce false memory in the DRM paradigm. In this paradigm, participants study several lists of related words (e.g., *bed*, *rest*, *tired*, etc.), each of which converges on a single nonstudied critical item (e.g., *sleep*). When tested, participants often recall and/or recognize the critical item as having been presented, resulting in a robust form of memory illusion. Researchers have subsequently explored various manipulations to reduce the DRM illusion, such as repeated study trials (Benjamin 2001; McDermott 1996), explicit warnings (Gallo et al. 2001; McCabe and Smith 2002), and—importantly for present purposes—distinctive encoding tasks (Arndt and Reder 2003; Dodson and Schacter 2001, 2002; Gunter et al. 2007; Huff and Bodner 2013; Israel and Schacter 1997; McCabe and Smith 2006). Distinctive encoding tasks may be particularly salutary because they can produce a *mirror effect* (Glanzer and Adams 1990)—increased correct memory coupled with decreased false memory—relative to a nondistinctive control condition (Gunter et al. 2007; Huff and Bodner 2013).

## Impoverished relational encoding versus the distinctiveness heuristic

The DRM literature, too, has been rife with debate over whether the benefits of distinctive encoding arise at encoding or at retrieval. The *impoverished relational encoding* account (Arndt and Reder 2003; Hege and Dodson 2004; Hockley and Cristi 1996) posits that distinctive processing reduces semantic activation of the critical items by decreasing their associations to list items (Roediger et al. 2001) or by reducing the thematic consistency of the lists (Brainerd and Reyna 2002). In contrast, the *distinctiveness heuristic* account postulates that distinctive effects are due to the adoption of a global diagnostic monitoring strategy at test (Schacter et al. 2001; Schacter et al. 1999). In this account, recollection of distinctive details at test provides diagnostic evidence that an item was studied, and this absence is diagnostic evidence that an item (including a critical item) was not studied.

Several approaches have been used to try to separate encoding and retrieval loci for distinctiveness effects in the DRM paradigm. The most common approach has been to test a *within-subjects condition*: Half the DRM lists are studied using a distinctive encoding method and half using a control (e.g., read-only) task. To review one example, Schacter et al. (1999) had participants study half the DRM lists with a picture of each list word’s referent provided and the other half without pictures. The DRM illusion was low and equivalent for both lists types, consistent with a retrieval-based distinctiveness heuristic (i.e., “if I can recall seeing the picture, the item is old; if not, it must be new”). Use of a global decision strategy increased monitoring and, thus, reduced false recognition of critical items from *both* distinctive and nondistinctive lists. In contrast, if the presentation of pictures at study had led to impoverished relational encoding, then the DRM illusion should have been selectively reduced for the distinctive (picture) lists. Although such results support the claim that distinctiveness benefits arise at retrieval, not all studies have found this pattern. For example, Arndt and Reder (2003) found a selective reduction in the DRM illusion for lists that were studied in distinctive (vs. nondistinctive) fonts, consistent with an encoding-based account.

Another approach to parsing out the contributions of encoding versus retrieval processes is through the use of an *inclusion test* (Brainerd et al. 2003; Gunter et al. 2007; Hege and Dodson 2004; Hunt et al. 2011). Under inclusion instructions, participants are asked to report or endorse all test items that were studied or are related to studied items. On this test, participants should therefore report or endorse the critical items, which presumably leads participants to abandon retrieval-based strategies such as the distinctiveness heuristic, that would otherwise operate to reduce the DRM illusion. As a result, any reduction of the DRM illusion due to distinctive encoding can be attributed to encoding-based processes. Similar to within-subjects tests, inclusion tests have yielded mixed findings. Distinctive encoding has sometimes reduced the DRM illusion, consistent with an encoding locus (e.g., Hege and Dodson 2004; Hunt et al. 2011), but other times has not, consistent with a retrieval locus (e.g., Gunter et al. 2007; Pierce et al. 2005; Schacter et al. 2001).

Most recently, we have advocated a *signal detection approach* for separating encoding and retrieval loci of distinctiveness effects in recognition (Gunter et al. 2007; Huff and Bodner 2013). Signal detection analyses attempt to partition participants’ underlying memory experiences for studied versus nonstudied items from their response bias, or proclivity to report at test that an item was studied. The theory assumes that recognition experiences map onto a continuum of strength and that the distribution of experiences are typically further along this continuum for studied items than for nonstudied items. The distance between the standardized mean of these hit and false alarm distributions, *d'*, provides an index of the amount of memory information that was encoded in a given condition (often termed sensitivity or discriminability; see MacMillan and Creelman 1991; Wickens 2002). Importantly, in the DRM paradigm, a *d'* index of memory information can also be calculated for false recognition of critical lures by calculating the standardized difference in recognition claims for critical items from studied lists (hits) minus nonstudied lists (false alarms). For false recognition, this analysis treats recognition claims for critical items from studied lists as hits and recognition claims for critical items from nonstudied lists as false alarms. A comparison of *d'* across distinctive and nondistinctive encoding conditions can thus be used to measure the encoding-based effects of distinctive encoding for both list items and critical items. For example, impoverished relational encoding should yield a smaller *d'* for critical items for distinctive (vs. nondistinctive) lists in a between design, whereas use of a distinctiveness heuristic should yield equivalent *d'*s.

Signal detection theory can also be used to derive a suitable index of response bias (i.e., how liberally or conservatively participants respond during test). In the DRM paradigm, we have suggested that more conservative responding is indicative of an increase in test-based strategic memory monitoring, consistent with use of a distinctiveness heuristic (Gunter et al. 2007; Huff and Bodner 2013). Traditional measures of response bias, such as criterion *c*, are measured using a hit rate and a false alarm rate. However, such measures are not appropriate when evaluating the contributions of distinctive encoding that can produce a mirror effect (an increase in hits and a decrease in false alarms) by moving the hits and false alarm distributions in opposing directions. Given this divergence, bias measures should not be computed using both hits and false alarms.

A solution to this issue was provided by a helpful anonymous reviewer who recommended that we use an index of response criterion, lambda (*λ*), which reflects the location of the nonstudied item distribution alone and, hence, is computed without recourse to a hit rate (see Wickens 2002). Lambda is computed as the *z*-score of 1 minus the false alarm rate. Higher *λ* values reflect a more conservative response bias that we interpret as greater retrieval-based monitoring. As with *d'*, *λ* can also be computed for both list items (on the basis of false alarms to list item from nonstudied lists—i.e., *list item controls*) and critical items (on the basis of false alarms to critical items from nonstudied lists—i.e., *critical item controls*). A comparison of *λ* across distinctive and nondistinctive encoding tasks can thus be used to gauge the retrieval-based effects of distinctive encoding for both list items and critical items. For example, use of a distinctiveness heuristic at test should yield larger *λ* values, whereas impoverished relational encoding should not affect *λ*.

Huff and Bodner (2013) argued that the signal detection approach offers advantages over other approaches for separating encoding and retrieval processes. In brief, comparing false recognition for distinctive and nondistinctive lists in a within design relies upon a null effect of list type to infer the operation of a retrieval-based distinctiveness heuristic. In addition, this design assumes that encoding and retrieval processes are mutually exclusive; therefore, it is unable to detect situations in which processes operate in tandem. Inclusion tests are similarly plagued: A null effect is again used to indicate use of a retrieval-based distinctiveness heuristic. Conclusions based on these tests alone result in the affirmation of a disjunction in which finding evidence for one process eliminates the contribution of the other process by default.

Using the signal detection approach, Huff and Bodner (2013) separated the contributions of encoding and retrieval processes to correct and false recognition in the DRM paradigm, but only across between-group designs. Across experiments, they compared the effects of pleasantness ratings, anagram generation, and processing instructions with those of nondistinctive (read-only) control groups. Their signal detection indices revealed an interplay between encoding and retrieval processes in modulating correct and false recognition. For correct recognition, the indices of memory information (*d'*) and of memory monitoring (*λ*) were both greater in the distinctive (vs. nondistinctive) groups, suggesting that the distinctiveness advantage was due to a combination of encoding and retrieval processes. For the DRM illusion, memory monitoring for critical items during test (i.e., the retrieval locus) was greater in the item-specific groups, but the effects on memory information (i.e., the encoding locus) differed across experiments. Specifically, memory information for critical items was lower following generation than following reading but was equivalent to reading following pleasantness ratings and item-specific instructions. Thus, distinctive encoding consistently increased retrieval-based monitoring, consistent with the distinctiveness heuristic account, but it led to impoverished relational encoding only following generation.^1^

## Distinctiveness effects in between versus within designs

The benefits of distinctive encoding on correct recognition have been well characterized in between and within designs (Begg et al. 1989; Bertsch et al. 2007; McCabe and Smith 2006). In a between (pure list) design, distinctive encoding tasks presumably induce item-specific processing that helps individuate the list items. In a within (mixed list) design, distinctive list items can be further contrasted to nondistinctive list items that receive less item-specific processing. Because of this additional relative contrast, the benefits of distinctive encoding are often greater in a within design than in a between design (Bertsch et al. 2007; Fawcett 2013; McDaniel and Bugg 2008). Alternatively, Begg and Snider (1987) argued that the robust (distinctive) generation effect in a within design may sometimes be due to cursory processing of the (nondistinctive) read items, rather than to enhanced memory for the generate items. In other words, the apparent benefits of distinctive processing in a within design may actually reflect costs to nondistinctive items.

The costs and benefits of distinctive encoding in a within design can be gauged by comparing each type of item/list with its pure list counterpart (e.g., Begg and Snider 1987; Bodner, Taikh, and Fawcett 2014). A benefit occurs when memory for distinctively encoded items is greater in a within (vs. between) design. A cost occurs when memory for nondistinctively encoded items is lower in a within (vs. between) design. Using this analysis, Begg and Snider concluded that the within-subject generation effect in recognition largely reflects a cost to (nondistinctive) read items rather than a benefit to (distinctive) generate items. More recently, Bodner, Taikh, and Fawcett assessed the costs and benefits of the within-subject production effect in recognition (i.e., an advantage for items studied aloud, as opposed to silently). The production effect is typically attributed to a benefit to aloud items resulting from increased distinctiveness (e.g., Bodner and Taikh 2012), yet their experiment and meta-analysis showed that, as with generation, the costs to silent (nondistinctive) items outweighed the benefits to aloud (distinctive) items.

An analysis of the costs/benefits of distinctiveness on both correct and false recognition in the DRM paradigm has never been conducted, and remedying this situation was our second key goal. As was discussed above, false recognition in within groups is typically equivalent for distinctive and nondistinctive lists, and these null effects have been marshaled as evidence of a global monitoring strategy at test (McCabe and Smith 2006; Schacter et al. 1999; cf. Arndt and Reder 2003). But to establish that the DRM illusion has been reduced in a within group, each list type must be compared with a pure list counterpart—comparisons that have not been made to date. Thus, the pattern of results in the within design may reflect benefits, costs, or a combination of the two. Here, benefits refer to a *decrease* in false recognition for critical items from nondistinctive (i.e., read) lists in the within group, relative to a pure-list read group. Costs refer to an *increase* in false recognition for critical items from distinctive lists in the within group, relative to a pure distinctive-list group.

Measuring the costs and benefits of distinctive processing in within (vs. between) designs is important because it can specify how distinctive encoding affects both correct memory and memory errors. Dodson and Schacter (2002) suggested that “there is no cost to using the distinctiveness heuristic” (p. 798) in the DRM paradigm, but they did not report comparisons across within and between designs to substantiate this claim. Our analyses determined whether distinctive encoding is truly “cost free” or might, for example, impair memory for nondistinctively encoded items. This is important in terms of recommending distinctiveness as a general strategy for memory improvement.

## Meta-analyses

We conducted a series of meta-analyses to accomplish two goals. First, we used signal detection indices to further evaluate Huff and Bodner’s (2013) claim that reductions in the DRM illusion due to distinctive encoding are driven by both encoding (impoverished relational encoding) and retrieval (distinctiveness heuristic) mechanisms. Second, we examined whether distinctive encoding exclusively produces benefits (i.e., a “cost free” strategy; Dodson and Schacter 2002) or might produce costs to nondistinctive items, as found in other domains (e.g., Begg and Snider 1987; Bodner, Huff et al. 2014). Furthermore, we evaluated how the effects of distinctive encoding are modulated by the use of a within (vs. between) design. That is, does the presence of nondistinctive lists in a within design affect the encoding and/or retrieval of the distinctive lists? In sum, we determined the costs and benefits of distinctive processing in each design, as well as their loci.

To accomplish these objectives, we searched for DRM recognition studies in which distinctive versus nondistinctive (i.e., read-only control) encoding tasks were manipulated across between and within designs in the same study (although usually across experiments). We searched for suitable studies via Web of Science, PsycInfo, and Google searches. Available authors of included studies were also emailed for other leads and suitable unpublished data sets. Distinctive encoding was operationalized as a task designed to improve memory for studied items and/or to decrease the DRM illusion. Studies that did not meet these requirements were excluded. Of the nine data sets we obtained that met these criteria, five used generation as the distinctive encoding condition:

1. Bodner, Huff, Gunter, and Azad (2014, unpublished raw data);
2. Gunter (2004, unpublished Master’s thesis);
3. Gunter et al. (2007, Experiment 1A vs. 2A);
4. Huff and Bodner (2014, unpublished raw data);
5. McCabe and Smith (2006, Experiment 1 vs. 2);

Three more studies presented pictures along with list words in the distinctive conditions:

6. Schacter et al. (1999, Experiment 1 vs. 2; younger adults);
7. Schacter et al. (1999, Experiment 1 vs. 2, older adults);
8. Schacter et al. (2001; Experiment 1).

The final study used production (i.e., saying list words aloud) in the distinctive conditions:

9. Dodson and Schacter (2001; Experiments 1 vs. 2).^2^

Discriminability (*d'*), an index of encoded memory information, and lambda (*λ*), an index of memory monitoring at test, were calculated from raw participant means, with the exception of McCabe and Smith (2006) and Schacter et al. (1999), for which the published group means and standard deviations were imputed from the weighted average of the other studies. Table 1 reports the mean indices for the distinctive and nondistinctive conditions in each study (Table 2 provides the mean hit and false alarm rates). The mean *d'* and *λ* scores were then used to calculate standardized mean difference scores for each of the comparisons described below. For between-group comparisons, these scores were calculated using the *escalc* function from the *metafor* package (Viechtbauer 2010) within R version 3.0.1 (R Development Core Team 2013). This function produces an estimate of Hedges’s *g* (Hedges 1981) corrected for its slight positive bias (Hedges and Olkin 1985). For within-group comparisons, comparable scores were calculated using the equations recommended by Borenstein (2009) to account for the dependence among the included means. The correlations necessary for these comparisons were calculated from the raw data where possible; for studies where this information was unavailable (McCabe and Smith 2002; Schacter et al. 1999), suitable correlations were imputed, instead, from the weighted average of the other studies (see, e.g., Higgins and Green 2009; Pigott 2009).^3^

**Table 1:** Mean signal detection indices of encoded memory information (*d*') and memory monitoring at test (*λ*) for between and within designs and for distinctive (D) and nondistinctive (ND) lists, and effects of design by list type and list type by design used in the meta-analyses

					Design Effect		List Effect	
	Between (B)		Within (W)		(B − W)		(Dlists − NDlists)	
Task/Study/Means	Dlists	NDlists	Dlists	NDlists	Dlists	NDlists	Between	Within
**Generation**								
Bodner, Huff, Gunter, & Azad (2014 **)** ^**b**^								
List item *d'*	2.56	1.13	2.65	1.92	−0.09	0.21	0.43	0.73
List item *λ*	1.47	1.34	1.37	0.10	−0.03	0.13		
Critical item *d'*	1.17	1.87	1.28	1.10	−0.11	0.77	−0.70	0.18
Critical item *λ*	1.18	1.06	1.11	0.07	−0.05	0.12		
Gunter (2004)								
List item *d'*	2.92	1.77	2.69	1.85	0.23	−0.08	1.15	0.84
List item *λ*	1.49	0.90	1.22	0.27	−0.32	0.59		
Critical item *d'*	0.93	1.27	1.10	0.85	−0.18	0.42	−0.34	−0.59
Critical item *λ*	1.09	0.54	0.79	0.30	−0.25	0.55		
Gunter, Bodner, & Azad (2007 **)**								
List item *d'*	2.76	2.21	2.81	1.91	−0.05	0.30	0.55	0.90
List item *λ*	1.62	1.32	1.27	0.35	0.05	0.30		
Critical item *d'* ^*a*^	1.09	1.43	1.10	0.93	−0.01	0.50	−0.34	0.17
Critical item *λ*	1.26	0.91	0.96	0.30	−0.05	0.36		
Huff and Bodner (2014)								
List item *d'*	2.73	2.11	2.61	1.53	0.12	0.58	0.62	1.08
List item *λ*	1.67	1.36	1.26	0.41	0.10	0.31		
Critical item *d'*	1.03	1.39	1.42	0.89	−0.39	0.41	−0.36	0.44
Critical item *λ*	1.34	1.02	1.11	0.23	−0.09	0.32		
McCabe and Smith (2006)^*^								
List item *d'*	2.62	2.27	2.32	1.89	0.30	0.38	0.35	0.43
List item *λ*	1.13	1.13	0.95	0.18	−0.18	0.00		
Critical item *d'*	1.35	2.01	1.99	2.05	−0.64	−0.04	−0.66	−0.06
Critical item *λ*	0.84	0.92	0.81	0.03	0.11	−0.08		
**Pictures**								
Schacter, Israel, & Racine (1999; younger adult group)^*^								
List item *d'*	2.11	1.61	1.91	1.63	0.20	−0.02	0.50	0.28
List item *λ*	1.34	0.81	0.95	0.39	−0.14	0.53		
Critical item *d'*	1.02	1.00	0.60	0.65	0.42	0.35	0.02	−0.05
Critical item *λ*	1.41	0.58	0.67	0.74	−0.13	0.83		
Schacter et al. (1999; older adult group)^*^								
List item *d'*	1.96	1.17	1.51	1.03	0.45	0.14	0.79	0.48
List item *λ*	1.14	0.58	0.77	0.64	−0.19	0.83		
Critical item *d'*	0.89	1.54	0.98	1.06	−0.09	0.48	−0.65	−0.08
Critical item *λ*	0.99	0.95	0.64	0.35	0.31	0.04		
Schacter, Cendan, Dodson, & Clifford (2001 **)**								
List item *d'*	1.99	1.68	2.11	1.88	−0.12	−0.20	0.31	0.33
List item *λ*	1.60	1.21	1.50	0.10	−0.29	0.39		
Critical item *d'*	0.65	1.12	0.66	0.66	−0.01	0.46	−0.46	0.00
Critical item *λ*	1.38	0.94	1.09	0.19	−0.15	0.44		
**Production**								
Dodson and Schacter (2001)								
List item *d'*	1.99	1.75	2.27	1.99	−0.28	−0.24	0.24	0.28
List item *λ*	1.22	1.15	1.38	−0.16	−0.23	0.07		
Critical item *d'*	0.73	1.38	1.34	1.31	−0.61	0.07	−0.65	0.03
Critical item *λ*	0.65	0.64	1.09	−0.44	−0.45	0.01		
Font								
Arndt and Reder (2003)^c^								
List item *d'*	2.49	2.78	2.67	2.76	−0.18	0.02	−0.29	−0.09
List item *λ*	1.27	1.43	1.18	0.09	0.25	−0.16		
Critical item *d'*	0.88	1.83	1.07	1.72	−0.19	0.11	−0.95	−0.65
Critical item *λ*	0.96	1.00	0.87	0.09	0.13	−0.04		
**Average** (excluding Arndt and Reder 2003 **)**								
List item *d'*	2.40	1.86	2.32	1.74	**0.08**	**0.12**	**0.55**	**0.58**
List item *λ*	1.44	1.09	1.19	0.25	−0.10	**0.35**		
Critical item *d'*	0.98	1.45	1.16	1.07	**−0.18**	**0.38**	**−0.46**	**0.10**
Critical item *λ*	1.13	0.84	0.92	0.21	−0.08	**0.29**		

*Note*. Bolded means correspond to effect sizes calculated in the meta-analyses. MD = mean difference as represented in the table.

* Signal detection indices were computed from published group means.

^a^Means reported in the original article were incorrect.

^b^Dlists collapsed across generation and self-imagery tasks.

^c^Dlists collapsed across the uncorrelated and unique font groups, NDlists is the correlated font group.

Following calculation of the effect sizes, a series of random-effects models were fitted to the aggregate data to estimate an overall effect for each comparison. All models were generated using the *rma* function from the *metafor* package in R (see Viechtbauer 2010). Although efforts were taken to avoid publication bias, trim and fill analyses (Duval 2005) were also conducted for each random-effects model. These models evaluate evidence for publication bias favoring the inclusion of small studies with large, statistically significant effect sizes, rather than small studies with small, statistically nonsignificant effect sizes. In the event that evidence exists for such a bias, the missing nonsupportive studies are imputed, and the model is reconducted to test the sensitivity of our analyses to those missing studies. Although typically used to gauge evidence for missing nonsupportive studies, we also evaluated evidence of missing supportive studies in the same manner. Except where specifically noted, the trim and fill analyses found no evidence of missing studies; thus, the resulting model did not differ from the reported models.

### Confirming the effects of distinctiveness

**Fig. 1:**
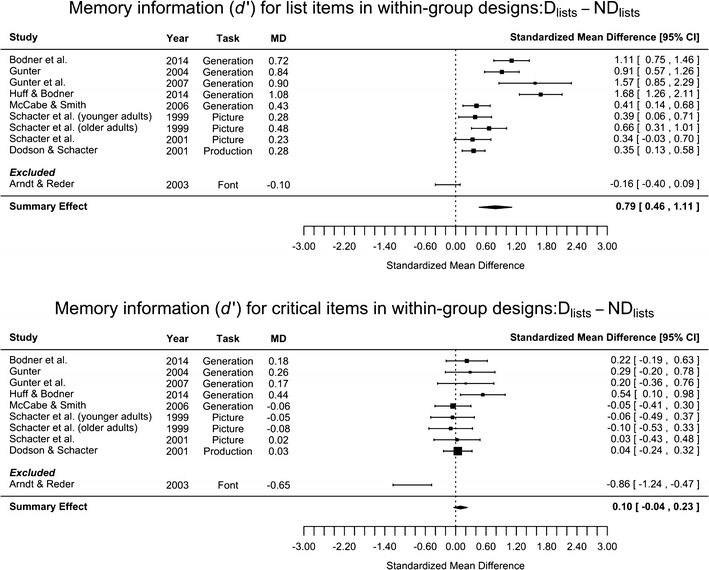
Meta-analyses of the influence of distinctive encoding on memory information for list items (top) and critical items (bottom) in within-subjects designs. Effect sizes and confidence intervals are based on standardized mean differences in a given index. The polygon at the bottom of each panel represents the summary effect for each analysis calculated using a random-effects model, excluding Arndt and Reder (2003; see Footnote 2). The square marker size indicates weight within the model. Dlists = distinctive lists; NDlists = nondistinctive lists. MD = mean difference as represented in Table 1

The first pair of meta-analyses (Fig. 1) sought to confirm the benefits of distinctive (vs. nondistinctive) encoding in the within-group “tests.” For studied list items, more memory information (*d'*) was encoded for distinctive (vs. nondistinctive) lists (*g* = 0.79, CI_95%_ = 0.46, 1.11; *Q*(8) = 53.07, *p* < .001; *I* ^2^ = 87.84%; Fig. 1, top). Importantly, there was no difference in encoded memory information (*d'*) for critical lures from distinctive versus nondistinctive lists (*g* = 0.10, CI_95%_ = −0.04, 0.23; *Q*(8) = 7.09, *p* = .527; *I* ^2^ < 0.01%; Fig. 1, bottom). The latter result confirms the distinctiveness pattern in which false recognition of critical items is equivalent for both list types in within-subjects groups.^4^

Inspection of Fig. 1 suggests that the presence of substantial heterogeneity in our analysis of memory information for studied list items was moderated by the type of distinctive encoding task. We therefore fit an exploratory mixed-effects model comparing data from generation tasks with the combination of all other distinctive tasks. This comparison was significant in a meta-regression model (*B* = 0.64, CI_95%_ = 0.13, 1.16), explaining 43.78% of the heterogeneity in our earlier model of these effects. The benefits were larger for generation (*g* = 1.07, CI_95%_ = 0.72, 1.43) than for the other distinctive tasks (*g* = 0.43, CI_95%_ = 0.06, 0.80).

### Do distinctiveness effects arise at encoding and/or retrieval?

**Fig. 2:**
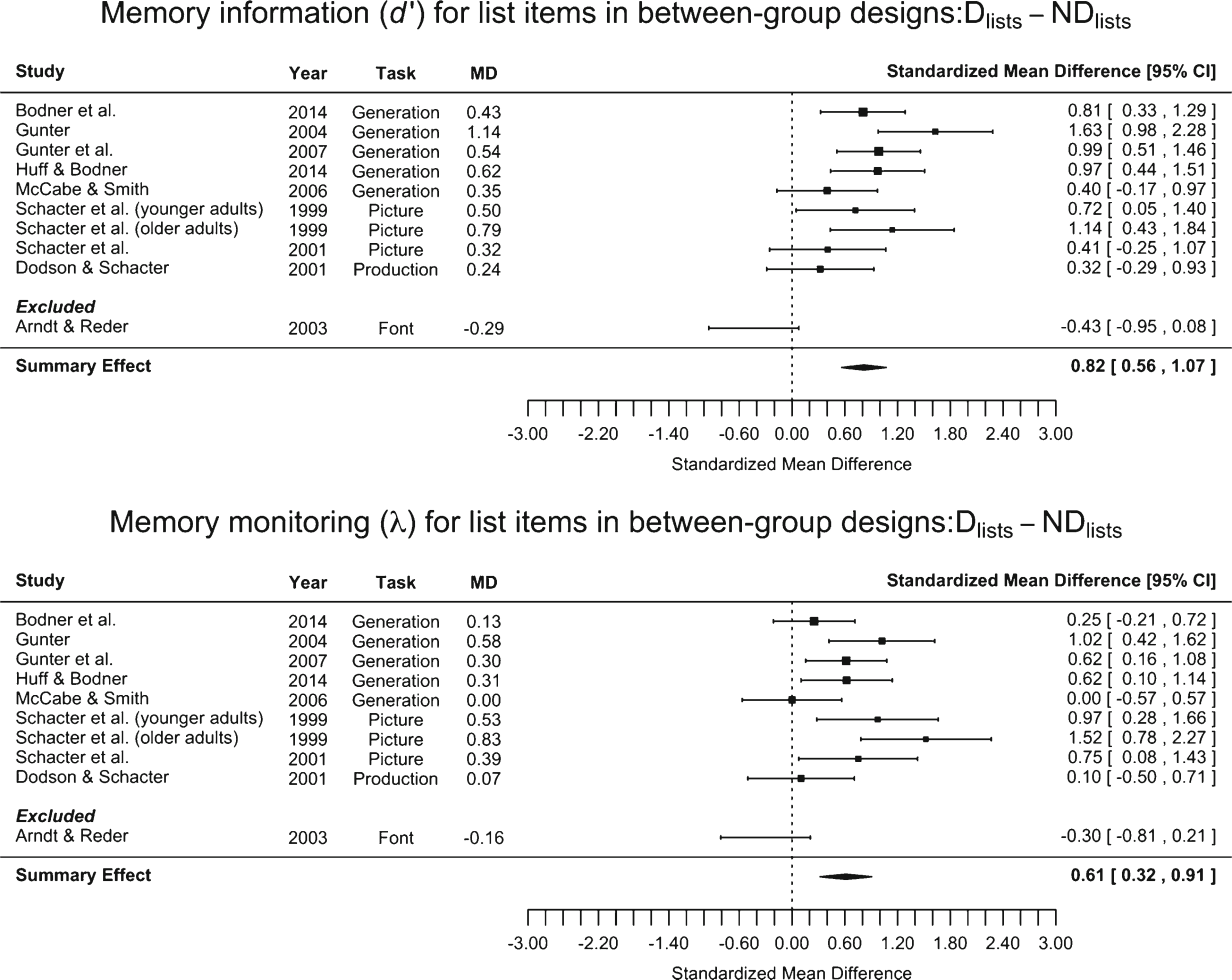
Meta-analyses of the influence of distinctive encoding on memory information (top) and memory monitoring at test (bottom) for list items in between-group designs. Effect sizes and confidence intervals are based on standardized mean differences in a given index. The polygon at the bottom of each panel represents the summary effect for each analysis calculated using a random-effects model, excluding Arndt and Reder (2003; see Footnote 2). The square marker size indicates weight within the model. Dlists = distinctive lists; NDlists = nondistinctive lists. MD = mean difference as represented in Table 1

The second pair of meta-analyses (Fig. 2) focused on correct recognition in the between-groups design and tested whether distinctive (vs. nondistinctive) encoding increases memory information and/or memory monitoring at test (*λ*) for list items. Distinctive (vs. nondistinctive) encoding increased encoded memory information (*d'*; *g* = 0.82, CI_95%_ = 0.56, 1.07; *Q*(8) = 13.75, *p* = .089; *I* ^2^ = 40.56%; Fig. 2, top) and also increased memory monitoring at test (*λ*; *g* = 0.61, CI_95%_ = 0.32, 0.91; *Q*(8) = 18.18, *p* = .019; *I* ^2^ = 57.06%; Fig. 2, bottom), replicating Huff and Bodner (2013). In the latter case, a trim-and-fill analysis revealed evidence of one missing study; however, imputing this missing study did not affect our conclusions (*g* = 0.53, CI_95%_ = 0.20, 0.86; *Q*(9) = 25.54, *p* = .019; *I* ^2^ = 67.69%). Thus, both encoding and retrieval processes facilitate recognition of list items following distinctive encoding.

**Fig. 3:**
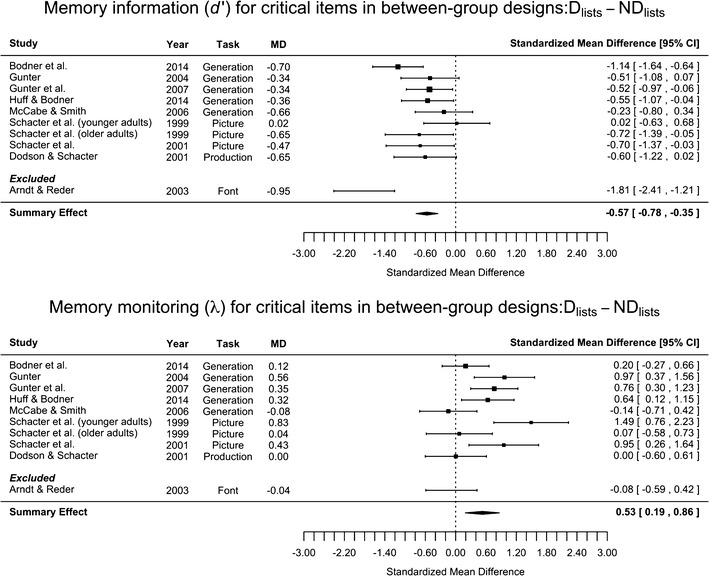
Meta-analyses of the influence of distinctive encoding on memory information (top) and memory monitoring at test (bottom) for critical items in between-group designs. Effect sizes and confidence intervals are based on standardized mean differences in a given index. The polygon at the bottom of each panel represents the summary effect for each analysis calculated using a random-effects model, excluding Arndt and Reder (2003; see Footnote 2). The square marker size indicates weight within the model. Dlists = distinctive lists; NDlists = nondistinctive lists. MD = mean difference as represented in Table 1

The third pair of meta-analyses (Fig. 3) were analogous, but focused on false recognition of critical items. The pattern here also replicated Huff and Bodner (2013): Distinctive groups encoded less memory information for critical items (*d'*; *g* = −0.57, CI_95%_ = −0.78, −0.35; *Q*(8) = 10.01, *p* = .265; *I* ^2^ = 22.49%; Fig. 3, top) and also performed more memory monitoring for critical items at test (*λ*; *g* = 0.53, CI_95%_ = 0.19, 0.86; *Q*(8) = 23.29, *p* = .003; *I* ^2^ = 67.47%; Fig. 3, bottom). These results provide compelling evidence that both encoding and retrieval factors shape distinctiveness benefits. Overall, the distinctive (vs. nondistinctive) groups encoded more information about list items and less information about critical items (consistent with an impoverished relational encoding account) and also performed more memory monitoring at test (consistent with a distinctiveness heuristic at test). Together, these influences induced a mirror effect: improved recognition of list items and reduced recognition of critical items.

### Do distinctiveness manipulations produce benefits and/or costs?

**Fig. 4:**
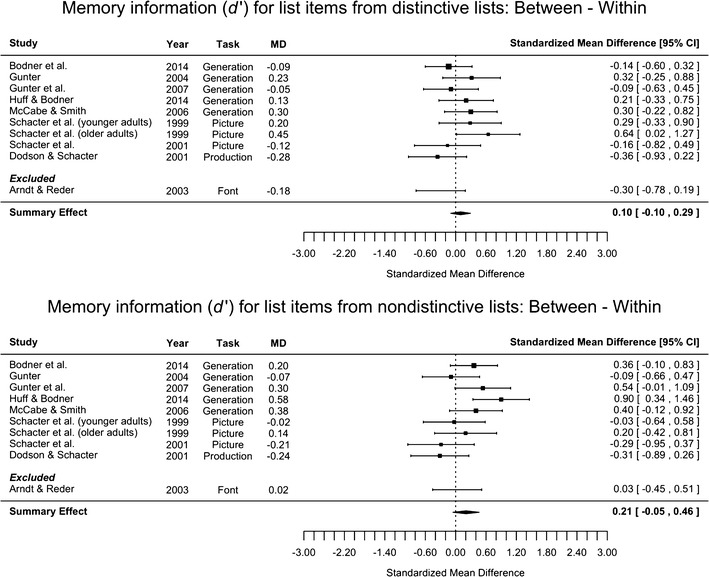
Meta-analyses of the influence of design on memory information for list items from distinctive lists (Dlists; top) and nondistinctive lists (NDlists; bottom). Effect sizes and confidence intervals are based on standardized mean differences in a given index. The polygon at the bottom of each panel represents the summary effect for each analysis, calculated using a random-effects model, excluding Arndt and Reder (2003; see Footnote 2). The square marker size indicates weight within the model. MD = mean difference as represented in Table 1

The fourth pair of meta-analyses (Fig. 4) evaluated whether the within (vs. between) groups showed improved recognition of distinctive list items and/or impaired recognition of nondistinctive list items. To gauge benefits, the *d'*s for distinctive lists were compared across between and within groups. To gauge costs, the *d'*s for nondistinctive lists were compared across the between and within groups. Recognition of distinctive list items was not improved in the within groups (*g* = 0.10, CI_95%_ = −0.10, 0.29; *Q*(8) = 9.09, *p* = .335; *I* ^2^ = 8.68% Fig. 4, top), nor was recognition of nondistinctive list items impaired in the within groups (*g* = 0.21, CI_95%_ = −0.05, 0.46; *Q*(8) = 15.21, *p* = .055; *I* ^2^ = 47.57% Fig. 4, bottom). In the latter case, a trim-and-fill analysis revealed evidence of one missing study that was *supportive* of the effect; in this case, imputing this missing study would result in a significant effect (*g* = 0.27, CI_95%_ = 0.001, 0.53; *Q*(9) = 19.46, *p* = .022; *I* ^2^ = 54.18%). Thus, distinctive encoding yielded neither benefits nor costs to correct recognition, contrary to prior demonstrations of costs to nondistinctive items (Begg and Snider 1987; Bodner, Taikh and Fawcett 2014).

However, an exploratory analysis revealed that the type of distinctive encoding task moderated costs. We fit a mixed-effects model comparing data from generation tasks with the combination of all other distinctive tasks. In the generation studies, recognition of nondistinctive list items suffered a cost in a within design (*g* = 0.42, CI_95%_ = 0.17, 0.67; Fig. 4, bottom). In contrast, there was no such cost to list item recognition for the other distinctive tasks (*d'*; *g* = −0.11, CI_95%_ = −0.43, 0.21). This difference was significant in a meta-regression model (*B* = 0.53, CI_95%_ = 0.12, 0.94), explaining 87.08% of the heterogeneity in that model.

**Fig. 5:**
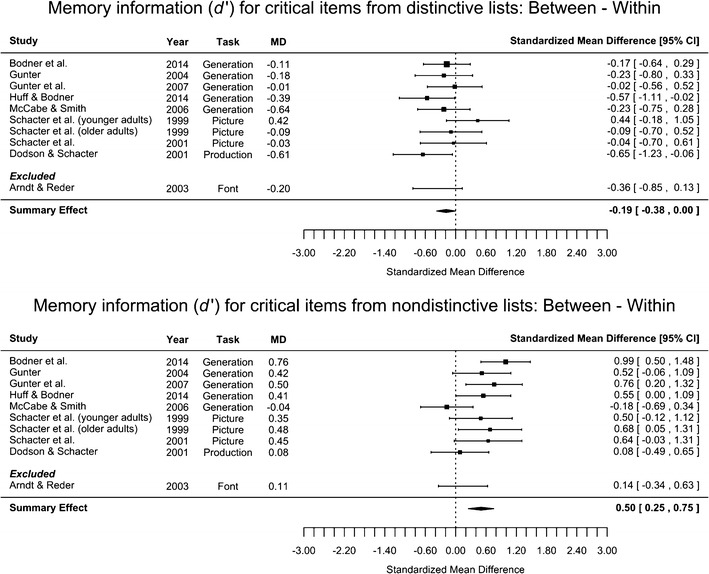
Meta analyses of the influence of design on memory information for critical items from distinctive lists (Dlists; top) and nondistinctive lists (NDlists; bottom). Effect sizes and confidence intervals are based on standardized mean differences in a given index. The polygon at the bottom of each panel represents the summary effect for each analysis, calculated using a random-effects model, excluding Arndt and Reder (2003; see Footnote 2). The square marker size indicates weight within the model. MD = mean difference as represented in Table 1

The final pair of meta-analyses (Fig. 5) evaluated the costs/benefits of distinctive encoding for false recognition of critical items. Interestingly, false recognition of critical items from distinctive lists was higher in a within (vs. between) design (*g* = −0.19, CI_95%_ = −0.38, 0.00, *p* < .05; *Q*(8) = 8.85, *p* = .355; *I* ^2^ = 0.26%; Fig. 5, top), showing a novel *distinctiveness cost*. Moreover, false recognition of critical items from nondistinctive lists was lower in a within (vs. between) design (*d'*; *g* = 0.50, CI_95%_ = 0.25, 0.75; *Q*(8) = 13.79, *p* = .087; *I* ^2^ = 43.10%; Fig. 5, bottom), showing a novel *nondistinctiveness benefit*. In this case, both analyses revealed evidence of two missing studies; in the former case, these missing studies were supportive of the effect, whereas in the latter case, they were nonsupportive; however, our conclusions remain unaffected for either the distinctiveness cost (*g* = −0.29, CI_95%_ = −0.50, −0.08; *Q*(10) = 16.13, *p* = .096; *I* ^2^ = 35.89%) or the nondistinctiveness benefit (*g* = 0.38, CI_95%_ = 0.12, 0.64; *Q*(10) = 23.58, *p* = .009; *I* ^2^ = 56.65%). Thus, on the one hand, a within design was less effective at reducing false recognition of distinctive critical items (i.e., it made the distinctive encoding task less effective—a cost), and on the other hand, it also reduced false recognition of critical items from nondistinctive lists (a benefit). Phrased differently, a within design is worse at reducing false recognition for distinctive lists but is better at reducing false recognition for nondistinctive lists.

## Discussion

The present review and meta-analyses helps bring clarity to the effects of distinctive encoding on recognition in the DRM paradigm and on the mechanisms that produce them. We examined whether distinctive encoding tasks improve correct recognition by facilitating encoding (amount of encoding memory information), retrieval (amount of memory monitoring at test), or both. Using signal detection estimates of each locus, the benefits of distinctive encoding of all DRM lists (i.e., in a between design) were shown to arise at both loci, as evidenced by increased *d'* and *λ*, respectively. Similarly, the benefits of distinctive processing on false recognition of critical items reflected a bifurcated pattern: a reduction in *d'* and an increase in *λ*. In other words, distinctive encoding tasks promote item-specific processing (and/or impair relational encoding) and also promote greater memory monitoring at test (i.e., a distinctiveness heuristic) in the DRM paradigm. Thus, our findings confirm that the two mechanisms are complementary rather than mutually exclusive (Huff and Bodner 2013).

We also evaluated whether the benefits of distinctive encoding in within designs reflect benefits and/or costs, by comparing each list type in the within design with its between group counterpart. For correct recognition, both types of list items yielded similar *d'*s across the two designs. However, when generation was the distinctive encoding task, we found that recognition of nondistinctively encoded list items suffered a cost in a within (vs. between) design, a pattern consistent with findings outside the DRM paradigm (e.g., Begg and Snider 1987; Bodner, Taikh and Fawcett 2014). This pattern raises the possibility that after encoding a generate list, participants engage in somewhat “lazier” encoding of a read list, as Begg and colleagues found for intermixed read and generate trials. If so, one peril of performing distinctive encoding for some lists is that it can lead to shallower encoding of nondistinctive lists. Whether this recognition trade-off is a pro or a con depends on the learner’s goals (see Bodner, Taikh and Fawcett 2014, for a discussion). If a learner’s goal is to increase correct recognition for a subset of studied items, distinctive processing of that subset of items will be effective. In other words, the cost/benefit trade-off is specific to a within-subjects design. However, if the learner wishes to improve recognition of all studied items, it may be better to engage in distinctive processing of all items.

For false recognition, distinctive encoding in a within design yielded both a cost and a benefit. The cost was that participants encoded more information about the critical items for the distinctively encoded lists. Thus, distinctive encoding was less effective at reducing the DRM illusion in a within design. Alternating encoding task type across lists may render the distinctive encoding task less effective; it may be worth exploring whether blocking distinctive and nondistinctive lists reduces this cost. The benefit was that participants encoded less information about the critical items for nondistinctively encoded lists in a within (vs. between) design. Performing distinctive encoding for some lists might have a carryover effect, either increasing item-specific encoding and/or decreasing relational encoding of nondistinctive lists (see Huff and Bodner 2013).

### Potential limitations of the meta-analyses

We believe there is merit to conducting meta-analyses even for small sets of studies to help bring resolution to existing debates (e.g., impoverished relational encoding vs. distinctiveness heuristic) and to reveal previously undetected effects (e.g., costs of distinctive encoding; see also Fawcett 2013). Of course, meta-analysis can also yield misleading effect size estimates, resulting in incorrect conclusions. We attempted to avoid the potential pitfalls of meta-analyses outlined by Rosenthal and Dimatteo (2002)*.*

First, we tried to avoid a publication bias by seeking and including unpublished data sets and by examining funnel plots for the presence of bias. These plots were not suggestive of publication bias—a fact also supported by our trim-and-fill analyses.

Second, the apples-to-oranges problem involves comparing data sets that used dissimilar independent and dependent variables. We avoided this pitfall by using only encoding tasks identified as “distinctive” in the literature and by analyzing means based on a single memory test (i.e., recognition). For this reason, we opted not to include studies that included between-group conditions but no within condition (e.g., Huff and Bodner 2013) when computing the between-group meta-analyses.

Third, nonindependence occurs when multiple data sets are taken from the same study. In our meta-analyses, this was true for two of our data sets (Schacter et al. 1999), but distinguishing them (rather than averaging them) proved important, as described below.

Fourth, we avoided the garbage-in/garbage-out problem by using peer-reviewed articles and/or unpublished data sets from studies using similar procedures/materials conducted in the same labs. Although the number of suitable studies was modest, it is not unprecedented (Bodner, Taikh and Fawcett 2014; Fawcett 2013). Moreover, this number was sufficient both for confirming known findings (see Fig. 3) and for establishing new findings as outlined above. Of course, it would be valuable to update the current meta-analyses once the available sample of studies dictates.

### Using signal detection indices to isolate encoding and retrieval effects

Using signal detection indices, we revisited previous evidence for the impoverished relational encoding and distinctiveness heuristic accounts. We confirmed that studies reporting evidence for use of a distinctiveness heuristic at test (Dodson and Schacter 2001; McCabe and Smith 2006; Schacter et al. 2001; Schacter et al. 1999) indeed yield higher levels of monitoring when signal detection analyses are applied, consistent with the authors’ original claims. Importantly, however, our analyses also revealed an encoding locus (in line with the impaired relational encoding claim) that was not detected in these individual studies.

To highlight one example of the merits of our signal detection approach, it prompts a reinterpretation of Schacter et al.’s (1999) findings. In their study, younger and older adults studied DRM lists in word and picture (distinctive condition) or word-only (nondistinctive) modalities in between and within designs. False recognition of critical items was reduced in the distinctive (vs. nondistinctive) group for both younger and older adults (see Table 2). Within subjects, false recognition was equivalently low for both list types for both age groups, leading the authors to conclude that the older adults benefited from a distinctiveness heuristic as much as younger adults.

Application of our signal detection analysis to the group level means in the between groups instead suggests a divergent pattern across the age groups.^5^ For younger adults, the distinctiveness heuristic account was upheld: Memory information (*d'*) for critical lures was similar across the distinctive and nondistinctive groups, and memory monitoring (*λ*) was numerically greater in the distinctive group. For older adults, however, signal detection analyses revealed the opposite pattern: Memory information for critical lures was numerically lower in the distinctive group, and memory monitoring was similar. In other words, the false memory reduction for older adults was due to encoding processes (e.g., impoverished relational encoding), rather than to an increase in memory monitoring at test. Although inconsistent with the conclusions drawn by Schacter et al. (1999), the older adult pattern revealed by our signal detection indices is more consistent with the memory and aging literature. Specifically, older adults often show deficits in source monitoring at test (Dywan and Jacoby 1990; Hashtroudi et al. 1990), which would likely also impair their ability to monitor diagnostic details at test and, hence, to benefit from use of a distinctiveness heuristic.

The signal detection approach was also used to gauge the costs/benefits of distinctive encoding in a within design (after Begg and Snider 1987; Bodner, Taikh and Fawcett 2014). Our analyses revealed that distinctive encoding can produce costs, in contrast to Dodson and Schacter’s (2002) surmise that a distinctiveness heuristic provides a cost-free memory strategy. Specifically, more memory information about critical items was encoded for distinctive lists in a within (vs. between) design. Given this finding, implementing distinctive encoding for all studied items may be more effective at reducing false recognition than implementing it on a subset of studied items. This pattern stands in curious opposition to the general finding that distinctive encoding tasks are often more effective in *within* designs (see McDaniel and Bugg 2008). Whether the DRM paradigm is anomalous in this respect becomes an interesting area for future research to explore.

Our signal detection analysis approach is not without potential drawbacks. Most obviously, the indices are offline, indirect estimates used to infer the presence of encoding and retrieval processes. In addition, using lambda as a quantitative metric of test-based monitoring is discordant with the qualitative aspects of monitoring that are assumed to occur when a distinctiveness heuristic strategy is applied at test. Also, our memory-monitoring measure can detect quantitative differences only in the efficacy of what participants monitor at test; it cannot determine which specific memorial detail or details they monitor. Of course, this criticism equally applies to other means used for contrasting encoding versus retrieval bases for distinctiveness effects—namely, the use of inclusion instructions and within designs (for a discussion, see Huff and Bodner 2013). Lambda will be unable to detect whether participants in different conditions monitor for different but equally diagnostic recollected details.

Despite these shortcomings, the ability to independently estimate encoding and retrieval processes is advantageous, and we advocate this approach for separating the encoding and retrieval contributions of other encoding tasks and in other memory paradigms. Powerful memory manipulations such as spacing (Balota et al. 2007; Crowder 1976; Glenberg 1977), retrieval practice (Roediger and Karpicke 2006), and survival processing (Nairne et al. 2007) have generated effects that have been attributed to encoding and/or retrieval processes. The relative contributions of each locus, at least in recognition tasks, can fruitfully be examined through signal detection analyses. Of note, these memory effects are also generally larger in within than in between designs, suggesting that both costs and benefits may contribute to the within effects. Determining how design type influences the utility of other encoding manipulations is worthy of research attention.

An additional question is whether encoding and retrieval processes as shown in recognition would also occur in other explicit memory tasks—most notably, free recall—especially if a generate–recognize process guides recall (Anderson and Bower 1972). There is reason to believe that distinctiveness effects may be smaller in recall than in recognition due to recognition’s greater sensitivity to item-specific encoding processes (McDaniel, Waddill, & Einstein, 1988). The challenge to separating encoding and retrieval processes in recall would be the inability to use lambda to assess monitoring at retrieval. Recall metrics such as clustering (Roenker et al. 1971) or conditional response probabilities (Kahana 1996) also do not provide independent estimates of encoding and retrieval parameters. Thus, important questions for future research include how to separate and assess the encoding and retrieval processes operating in recall and whether recall produces similar patterns of costs and benefits across between and within designs.

## Conclusion

We suggest that meta-analysis of signal-detection indices provides a useful tool for examining encoding and retrieval processes and their effects on correct and false recognition. Our analyses revealed that reductions in the DRM illusion following distinctive encoding arise from a combination of encoding processes (i.e., reduced relational processing at study) and retrieval processes (i.e., increased strategic monitoring for critical items at test). Furthermore, use of a within (vs. between) design was found not to be “cost free” after all; although it reduced false recognition for nondistinctive lists, it inflated false recognition for distinctive lists. Researchers therefore need to exercise caution in extrapolating the results of particular study designs to general recommendations for encoding strategies for learners.

## Appendix

**Table 2:** Recognition means for between and within designs and for distinctive (D) and nondistinctive (ND) lists, and effects of design by list type and list type by design

	Between (B)		Within (W)		Design Effect (B − W)		List Effect (Dlists − NDlists)	
Task/Study/Means	Dlists	NDlists	Dlists	NDlists	Dlists	NDlists	Between	Within
**Generation**								
Bodner, Huff, Gunter, & Azad (2014 **)** ^a^								
List items	.85	.77	.88	.69	−.03	.07	.09	.19
List item controls	.09	.12	.11	−.03	.01	−.03		
Critical items	.49	.68	.56	.51	−.07	.17	−.19	.05
Critical item controls	.15	.18	.16	−.02	.02	−.03		
Gunter (2004)								
List items	.90	.79	.93	.69	−.03	.10	.11	.24
List item controls	.10	.21	.13	−.03	.08	−.11		
Critical items	.45	.75	.56	.51	−.11	.24	.30	−.05
Critical item controls	.15	.32	.25	−.10	.07	−.17		
Gunter, Bodner, & Azad (2007 **)**								
List items	.85	.79	.92	.70	−.07	.09	.06	.22
List item controls	.07	.12	.12	−.05	.00	−.05		
Critical items	.44	.68	.55	.49	−.11	.17	−.24	.06
Critical item controls	.10	.20	.19	−.08	.02	−.10		
Huff and Bodner (2014)								
List items	.84	.75	.90	.60	−.06	.15	.09	.30
List item controls	.06	.11	.12	−.06	−.01	−.05		
Critical items	.39	.63	.60	.46	−.21	.17	−.24	.14
Critical item controls	.09	.17	.13	−.04	.04	−.08		
McCabe and Smith (2006)								
List items	.88	.82	.85	.76	.03	.06	.06	.09
List item controls	.13	.13	.17	−.04	−.04	.00		
Critical items	.59	.76	.73	.75	−.14	.01	−.17	−.02
Critical item controls	.20	.18	.21	−.01	−.03	.02		
**Pictures**								
Schacter, Israel, & Racine (1999; younger adult group)								
List items	.78	.79	.83	.75	.05	.04	−.01	.08
List item controls	.09	.21	.17	−.08	.04	−.12		
Critical items	.35	.66	.47	.49	−.12	.17	−.31	.02
Critical item controls	.08	.28	.25	−.17	.03	−.20		
Schacter et al. (1999; older adult group)								
List items	.71	.72	.77	.60	−.06	.12	−.01	.17
List item controls	.08	.28	.22	−.14	.06	−.20		
Critical items	.46	.72	.63	.66	−.17	.06	−.26	−.03
Critical item controls	.16	.17	.26	−.10	−.09	−.01		
Schacter, Cendan, Dodson, & Clifford (2001 **)**								
List items	.64	.67	.70	.62	−.06	.05	−.03	.08
List item controls	.05	.14	.08	−.03	.06	−.09		
Critical items	.27	.56	.36	.37	−.09	.19	−.29	−.01
Critical item controls	.03	.18	.16	−.13	.02	−.15		
**Production**								
Dodson and Schacter (2001)								
List items	.76	.71	.79	.70	−.03	.01	.05	.09
List item controls	.16	.16	.12	−.04	−.04	.00		
Critical items	.51	.72	.58	.57	−.07	.15	−.21	.01
Critical item controls	.28	.29	.14	.14	−.15	−.01		
Font								
Arndt and Reder (2003)^b^								
List items	.87	.89	.90	.93	−.03	−.04	−.02	−.03
List item controls	.12	.10	.14	−.02	−.04	.02		
Critical items	.48	.77	.54	.77	−.06	.00	−.29	−.23
Critical item controls	.20	.19	.19	.01	.00	.01		
**Average** (excluding Arndt and Reder 2003 **)**								
List items	.80	.76	.84	.68	−.04	.07	.04	.16
List item controls	.09	.16	.14	−.05	.03	−.07		
Critical items	.44	.68	.56	.53	−.12	.15	−.25	.03
Critical item controls	.14	.22	.19	−.06	.02	−.08		

^a^Dlists collapsed across generation and self-imagery tasks.

^b^Dlists collapsed across the uncorrelated and unique font groups; NDlists is the correlated font group.

## Footnotes

1 Gunter et al. (2007) reported that generation increased memory monitoring but did not decrease memory information encoded for critical items. However, in preparing our meta-analyses, we discovered an error in their data coding that led to an underestimation of the latter effect. In fact, generation reduced memory information encoded for critical items in their study, as in Huff and Bodner (2013). Table 1 provides the corrected means for Gunter et al.

2 Arndt and Reder (2003) manipulated distinctive versus nondistinctive fonts across between and within designs in the DRM paradigm. This study was initially included in our meta-analyses, but it was often identified as an outlier by visual inspection and regression-deletion diagnostics (e.g., Cook’s distance, studentized deleted residual). Calculation of the covariance ratio further suggested that its inclusion substantially reduced the precision of our estimated effects. Therefore, this study was included in the tables and meta-analysis figures for comparison purposes but was eliminated from the summary effects in the meta-analyses. The possibility that font (a stimulus-based, perceptual manipulation) might differ in the basis of its effect from other distinctiveness tasks (processing-based, conceptual manipulations) warrants further exploration.

3 Sensitivity analyses carried out on these and other imputations reported verify that our findings are robust across the range of possible values observed for each variable.

4 Meta-analyses of memory monitoring across distinctive and nondistinctive lists in the within groups was not possible, because critical item controls do not belong to either distinctive or nondistinctive lists.

5 These indices were based on the published group-level means and, hence, could not be analyzed. As a result, these numerical patterns should be interpreted with caution.
